# Natural Products as Sources of Antimalarial Drugs

**DOI:** 10.1155/2020/9385125

**Published:** 2020-04-07

**Authors:** Philip F. Uzor, Vivitri D. Prasasty, Chukwuma O. Agubata

**Affiliations:** ^1^Department of Pharmaceutical and Medicinal Chemistry, University of Nigeria, Nsukka, Nigeria; ^2^Faculty of Biotechnology, Atma Jaya Catholic University of Indonesia, Jakarta, Indonesia; ^3^Department of Pharmaceutical Technology and Industrial Pharmacy, University of Nigeria, Nsukka, Nigeria

Malaria remains a devastating disease in several parts of the world, particularly in sub-Saharan Africa. A number of drugs have been developed over the years for the treatment of the disease, but the newly introduced artemisinin-based combination therapy (ACT) is the main stay drug used today. There is presently a deep concern that the Plasmodium parasite will soon develop total resistance to the existing antimalarial drugs due to the recent reports of increasing resistance of the parasite to the ACT, in many endemic areas. In addition, the mosquito vector has also developed resistance against the insecticides. The high cost as well as the adverse effects associated with some of the existing drugs also limits their usefulness in malarial treatment. Given the above, there is currently an urgent need to search for novel and more efficacious drugs to combat the disease. Natural products, including medicinal plants, have been used in the traditional treatment of malaria for thousands of years due to their efficacy, safety, lower cost, and availability. In fact, the two most successful antimalarial drugs, artemisinin and quinine, were sourced from medicinal plants. In this special issue, we intend to focus on recent studies dealing with the antimalarial activity of natural products (including extracts and compounds) derived from medicinal plants, animals, or microorganisms. Articles on the use of herbs in combination with drugs for the treatment of malaria also fall within the scope of this special issue. A brief summary of all accepted papers is provided as follows.

In a review paper by P. F. Uzor, the structures, activities, and toxicities of alkaloids which were isolated recently (2013 to 2019) from medicinal plants and reported to exhibit antiplasmodial activity were presented. Several classes of alkaloids, including terpenoidal, indole, bisindole, quinolone, and isoquinoline alkaloids, were identified with a promising antimalarial activity. It is hoped that the outcome of the review work will spur further research into the structural modification and/or development of the interesting compounds as novel antimalarial drugs.

D. Okello and Y. Kang carried out a comprehensive electronic review of various plant resources used to treat malaria across communities in Uganda. Approximately, 182 plant species from 63 different plant families were found to be used for malaria treatment in Uganda, of which 112 plant species have been investigated for antimalarial activity and 96% of the plant species showed positive results. These plants could be potential sources for potent antimalarial remedies.

A paper by A. Widyawaruyanti et al. investigated the effect of formulated *Artocarpus champeden* stem bark extract on parasite growth and immune response of *Plasmodium berghei*-infected mice in order to explain the mechanism for the antimalarial activity of the plant. The parasitemia, survival time, liver enzymes, and cytokine Th1 (IFN-*γ*, TNF-*α*) and Th2 (IL-10) levels were determined in infected mice. Results revealed that the plant extract was effective in inhibiting *P. berghei* growth and extending survival time. No effect on liver function was observed. The antimalarial effect of the plant could be explained in part by the enhanced production of the proinflammation cytokines, IFN-*γ*, and TNF-*α*.

A paper by R. Larayetan et al. studied the *in vitro* antimicrobial, antitrypanosomal, antimalarial, and antioxidant potentials of the crude leaf extracts (ethyl acetate and methanolic) of *Callistemon citrinus.* The phytochemical constituents were assessed by gas chromatography-mass spectrometry (GC-MS). Both extracts were bactericidal to *Escherichia coli* and *Pseudomonas aeruginosa*. They exhibited antitrypanosomal potentials with IC_50_ of 6.6/9.7 *μ*g/mL and antiplasmodial activities with IC_50_ of 8.4/13.0 *μ*g/mL as well as antioxidant potentials. Both were characterized by high amount of fatty acids (52.88 and 62.48%).

In a paper by O. A. Idris et al., the authors reported the *in vitro* bioactivities of *Rumex crispus* L. leaf and root extracts using toxicity, antimicrobial, and antiparasite assays. The study revealed that *Rumex crispus* has potency against microorganisms, Trypanosoma and Plasmodium, and could be a potential source for the treatment of these diseases.

W. Ekasari et al. reported the antimalarial activity of various parts of *Helianthus annuus* in both *in vitro* and *in vivo* assays. Inhibition of heme detoxification was also evaluated by the *β*-hematin level. The study revealed that the ethanol extracts of root of *H. annuus* showed promise as a new source for the development of new plant-based antimalarial agent.

Z. O. Ibraheem et al., in their paper, examined the *in vitro* antiplasmodium and chloroquine resistance reversal effects of andrographolide (a natural compound originally isolated from *Andrographis paniculata*). *In silico* molecular characterization was done using the Molinspiration software. Results showed that andrographolide has a moderate antiplasmodium effect and chloroquine resistance reversal effect which may be due to inhibition of chloroquine accumulation or due to its impact on the biological activity of the parasite. The study suggests the addition of andrographolide with chloroquine for reversal of chloroquine resistance and tolerance, but further *in vivo* studies are recommended to confirm this notion.

In a paper by D. Z. Wondafrash et al., the antimalarial activity of *Cordia Africana* (Lam.) (Boraginaceae) leaf extract and solvent fractions was investigated in *P. berghei*-infected mice. The study indicated that both the extract and fractions possess antimalarial effect.

*Philip F. Uzor* *Vivitri D. Prasasty* *Chukwuma O. Agubata*

